# Drivers of cardiovascular disease in metabolic dysfunction-associated steatotic liver disease: the threats of oxidative stress

**DOI:** 10.3389/fcvm.2024.1469492

**Published:** 2024-10-01

**Authors:** Erika T. Minetti, Naomi M. Hamburg, Reiko Matsui

**Affiliations:** Whitaker Cardiovascular Institute, Section of Vascular Biology, Boston University Chobanian & Avedisian School of Medicine, Boston, MA, United States

**Keywords:** cardiovascular risk, steatosis, MASLD, liver sinusoid, insulin resistance, glutathione, oxidative stress, remdesivir

## Abstract

Non-alcoholic fatty liver disease (NAFLD), now known as metabolic-associated steatotic liver disease (MASLD), is the most common liver disease worldwide, with a prevalence of 38%. In these patients, cardiovascular disease (CVD) is the number one cause of mortality rather than liver disease. Liver abnormalities *per se* due to MASLD contribute to risk factors such as dyslipidemia and obesity and increase CVD incidents. In this review we discuss hepatic pathophysiological changes the liver of MASLD leading to cardiovascular risks, including liver sinusoidal endothelial cells, insulin resistance, and oxidative stress with a focus on glutathione metabolism and function. In an era where there is an increasingly robust recognition of what causes CVD, such as the factors included by the American Heart Association in the recently developed PREVENT equation, the inclusion of liver disease may open doors to how we approach treatment for MASLD patients who are at risk of CVD.

## Introduction

**Figure 1:**
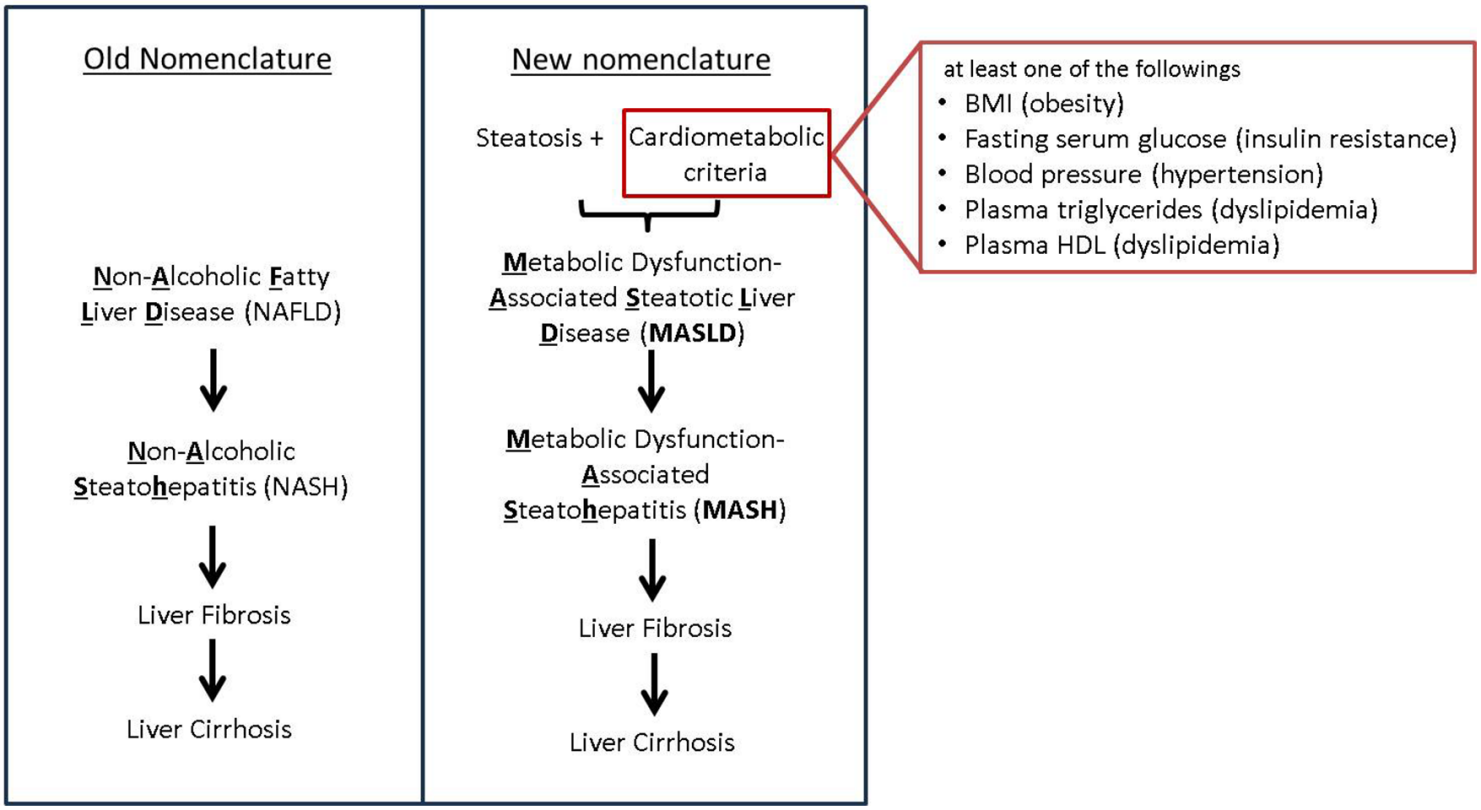
New nomenclature and criteria. Transition from the old NAFLD nomenclature to the new MASLD terminology, highlighting key differences in diagnostic criteria. The old NAFLD criteria focused primarily on hepatic steatosis whereas the new MASLD criteria take into account a broader range of metabolic risk factors.

Non-alcoholic fatty liver disease (NAFLD) is the most common chronic liver disease in the United States and its incidence is associated with obesity and diabetes (1). In 2023, there has been a change in nomenclature, from NAFLD to MASLD (Metabolic dysfunction-Associated Steatotic Liver Disease) following a multi-society Delphi decision to use a more descriptive and less stigmatizing disease name (2). Following this change, we will be using the most updated nomenclature in our review paper. However, the new nomenclature is accompanied also by a change in criteria. (Figure 1) In spite of the difference of criteria, discrepancy in population between NAFLD and MASLD is minimal. Our knowledge of NAFLD can be still valid in the context of MASLD (3). As such, in this paper we will use the term MASLD/MASH (Metabolic Dysfunction-Associated Steatohepatitis) while discussing findings from papers that studied NAFLD and/or NASH.

Fatty liver or steatosis alone is not necessarily harmful but may progress to metabolic dysfunction associated steatohepatitis (MASH) with inflammation and fibrosis. People with MASH have a higher risk of developing liver cirrhosis and hepatocellular carcinoma. The presence of fibrosis increases severity of the disease and extrahepatic complications. MASLD with steatohepatitis is designated as MASH (2).

Recently, MASLD has been recognized as an independent risk factor of cardiovascular diseases (CVD) (4, 5). Clinical studies reported that the death of MASLD patients was caused by CVD more than by liver-related disease (1, 6, 7). MASLD was also associated with a higher risk of atherosclerosis (8, 9), hypertension (10, 11), valvular heart disease, cardiomyopathy, and arrhythmias (12, 13). It may not be surprising to find the link because MASLD is associated with common metabolic risk factors including obesity, hyperlipidemia, and diabetes, which have cardiovascular implications. Extensive reviews regarding MASLD and its cardiovascular or extrahepatic complications are available elsewhere (4, 6, 14, 15). In this review, we discuss the mechanistic connection between MASLD and CVD besides epidemiological associations, with a focus on oxidative stress (oxidants) and insulin resistance. Oxidants contribute to MASLD progression and are known to exacerbate CVD. However, simple antioxidant therapies do not result in significant effects either on MASLD or CVD. Elucidation on the connection between MASLD and CVD may aid to prevent CVD in people with MASLD.

## Epidemiology

The global prevalence of MASLD in 2016 was estimated to be 37.8%, with numbers higher in men (39.7%) than women (25.6%) (16, 17) approximately a quarter of adults in the U.S. have MASLD (17).

A retrospective study of MASLD patients found that CVD was the number one cause of mortality, accounting for almost half the reported deaths, followed by malignancies (18%) and liver-related disease (18). MASLD is associated with high incidence of myocardial infarctions, atrial fibrillation, and cardiomyopathy, linked to peripheral arterial disease, with also some controversial evidence on the association with strokes (19–26). Fibrosis, in particular, is a predictor of CV disease and severity as it is associated with atrial fibrillation, myocardial infarctions and strokes (12, 24, 27, 28).

MASLD is defined as steatotic liver disease with at least one of five conditions including impaired lipid, glucose metabolism, and hypertension (Figure 1). It is well known that MASLD has shared risk factors such as obesity and diabetes with CVD.

Obesity is a characterizing factor in the development of MASLD, as body mass index (BMI), waist circumference, and body fat mass are all significantly correlated with elevated risk for MASLD (29). Steatosis was found correlating to degree of obesity (30). However, a meta-analysis reports that 25% of people with MASLD are lean, and cardiovascular cause of death was similar in lean MASLD compared with obese MASLD (31). Therefore, obesity is associated with MASLD but may not directly cause CVD in MASLD patients.

T2DM is also a significant risk factor for MASLD, as two thirds of T2DM patients have MASLD, with about one third having liver fibrosis (32). Diabetic patients with MASLD have higher risk of CVD than those without MASLD, suggesting syn**e**rgic effects of diabetes and MASLD. Anti-diabetic drugs have been beneficial effects on MASLD/MASH in diabetic patients. Glucose-lowering drugs improve hepatic function and steatosis, but the effects on liver fibrosis is questionable. It is still under investigation if anti-diabetic drugs work on MASLD without diabetes (33–35).

## Hepatic pathology in MASLD

### Liver sinusoidal endothelial cells in MASLD

The hallmark of MASLD is hepatic fat accumulation, and the underlying mechanisms of this process have been shown to drive CVD. The liver acquires lipids by uptake of fatty acids and via *de novo* lipogenesis, while it disposes lipids by fatty acid oxidation and by exporting as very low-density lipoprotein (VLDL). When hepatic lipid acquisition exceeds disposal, it results in accumulation of hepatic fats (36).

At cellular level in the liver, lipotoxicity due to excess lipids triggers hepatocyte death, Kupffer cells and immune cells activation, and increased inflammatory molecules. The liver has unique liver sinusoidal endothelial cells (LSEC) that cover hepatic sinusoids between blood and hepatocytes to facilitate the exchange of macromolecules through distinct fenestrae. LSECs play a critical role in filtration, vascular tone, immune response, and endocytosis among other functions (37, 38). Lipotoxicity and inflammation may cause capillarization (differentiation) of LSECs, a process whereby LSECs lose the normal structure of their fenestrae, leading to molecular transport dysfunction.

A study in mice showed that LSEC capillarization occurs in early stages in MASLD and precedes the activation of Kupffer cells and Hepatic stellate cells (HSCs). Researchers detected morphological changes in size and number of LSEC fenestrae, and increased expression of CD31 and CD34, both indicators of capillarization (39).

In a mouse model where LSEC fenestrae formation is impaired due to plasmalemma vesicle-associated protein deficiency, the mice developed multiple hallmarks of MASLD such as steatosis, hepatocyte ballooning, infiltration of macrophages, and collagen production by HSCs (40). Impaired fenestrae in LSEC in this mouse also causes higher plasma levels of LDL, cholesterol, triglycerides, and lower HDL level, showing the impact on LSEC capillarization on CV health (40).

**Figure 2:**
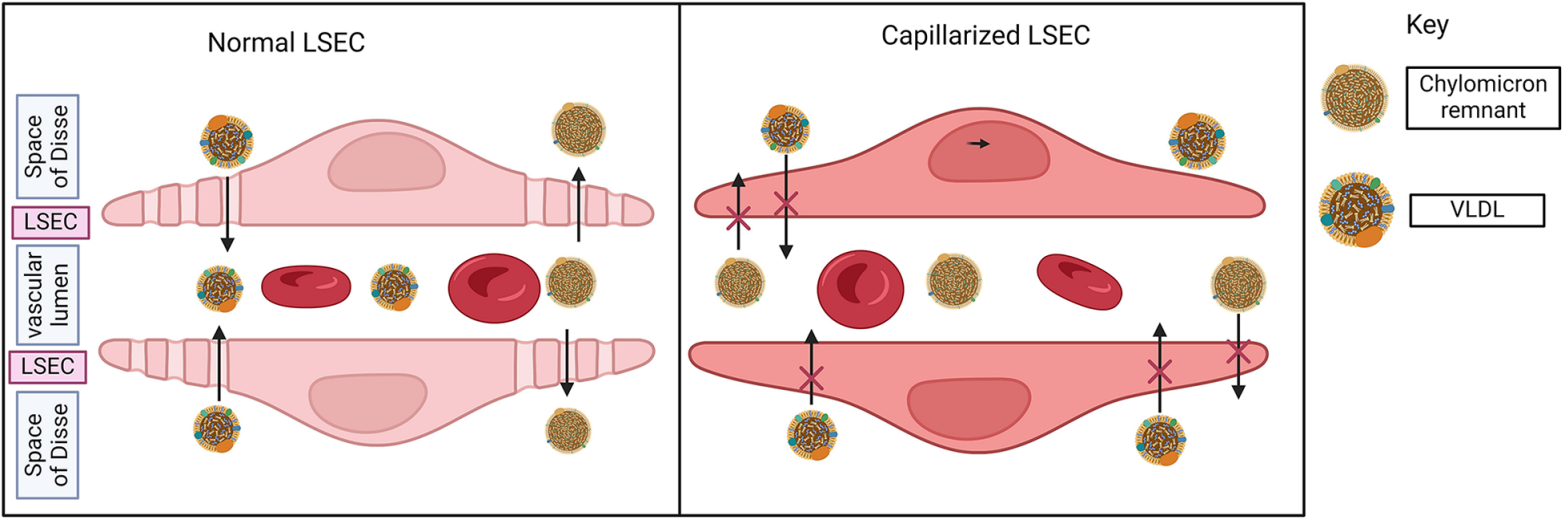
Liver sinusoidal endothelial cells (LSECs) in MASLD. Normal LSEC has a unique fenestrae by which macromolecules may transfer between the bloodstream and space of Disse (space between hepatocytes and sinusoid). LSEC reduces fenestrae and differentiates or capillarizes in the liver of MASLD. In the presence of normal LSEC, triglyceride-rich chylomicron remnants are removed from the bloodstream (vascular lumen → fenestrae → space of Disse). Similarly, VLDL enters the bloodstream via LSEC fenestrae. With the loss of fenestrae, both of these processes are impaired, driving atherosclerosis and hepatic steatosis respectively.

LSEC capillarization leads to atherosclerotic cardiovascular disease in particular by changes in the dynamics of lipid transport. Loss of LSEC fenestrae disrupts the uptake of lipids such as triglyceride-rich chylomicron remnants from blood into the space of Disse and then by hepatocytes. The reduced hepatic ability to remove the triglyceride-rich chylomicron remnants from blood causes hyperlipidemia, and an independent risk factor of atherosclerotic CVD (41). Loss of fenestrae also disrupts lipid transport within the liver by trapping VLDL and driving steatosis (42, 43) (Figure 2).

In addition, the liver uptakes majority of gut microbiota-derived lipopolysaccharides (LPS) through LSEC fenestrae. Therefore, it is hypothesized that serum lipopolysaccharides (LPS) levels may be increased in MASLD because hepatic LPS clearance is impaired (44). Low-level increase in circulatory LPS aggravates plaques formation (45), promoting atherosclerosis.

Patients with MASLD have vascular endothelial dysfunction as assessed by flow-mediated dilation (46). LSECs also show impairments in NO-mediated relaxation, a hallmark of endothelial dysfunction, causing impaired microcirculation in the liver (47). In addition, VEGF-induced fenestration of LSECs requires NO (48). The diminished production of NO in the dysfunctional LSEC could thus be affecting the VEGF-dependent maintenance of LSEC fenestrae, leading to capillarization.

### Insulin resistance in MASLD

To understand how insulin resistance contributes to the development of CVD in the context of MASLD, it is important to understand which tissues progressively become more resistant to insulin and how they contribute to the manifestations of MASLD. Insulin resistance in skeletal muscle decreases glycogen synthesis in the muscle, and increases hepatic *de novo* lipogenesis and triglyceride synthesis, resulting in atherosclerotic dyslipidemia in lean insulin resistant people (49). Consequently, the liver uptakes and accumulates lipids, affects insulin signaling, and causes hepatic insulin resistance (50).

**Figure 3:**
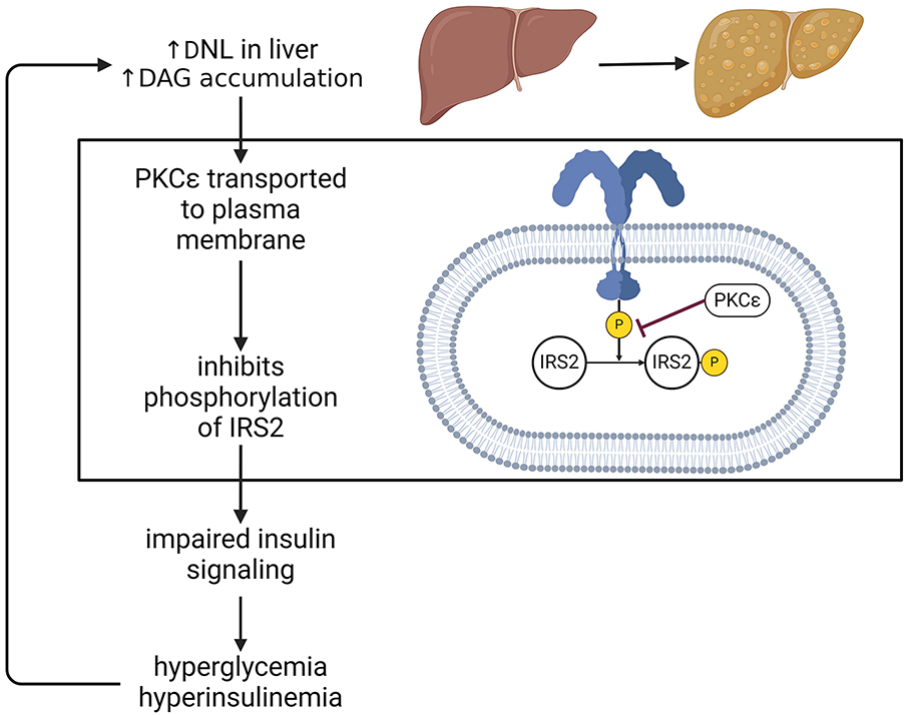
Insulin resistance in MASLD. One major consequence of steatosis is hepatic insulin resistance. In one of the mechanism, this can occur via PKCε mediated inhibition of IRS2 phosphorylation, thus dampening the effects of insulin. This impairment drives hyperglycemia and hyperinsulinemia, which in turn stimulate *de novo* lipogenesis, exacerbate fat accumulation in the liver and perpetuate a detrimental cycle affecting systemic metabolism. DNL, *de novo* lipogenesis; PKC, protein kinase C; DAG, diacylglycerol; IRS, insulin receptor substrate.

Elevated *de novo* lipogenesis causes an accumulation of diacylglycerols in the liver, which are an intermediate in the biosynthesis of triacylglycerols. An increase in diacylglycerols causes protein kinase-C*ε* (PKC*ε*) to be transported to the plasma membrane where it binds to the kinase portion of the insulin receptor, and inhibits phosphorylation of insulin receptor substrate 2 (IRS2), resulting in a diminished response to insulin. Insulin resistance results in elevated plasma glucose and insulin levels. Insulin activates enzymes that are involved in fatty acid synthesis. Thus, increased plasma insulin promotes hepatic *de novo* lipogenesis and triglycerides synthesis, unsuppresses gluconeogenesis in the liver, as well as impairs glycogen synthesis (51–53) (Figure 3).

Triglycerides are normally exported from the liver in VLDL particles, which contain apolipoprotein B100 (apo B100), and this apolipoprotein formation is facilitated by the enzyme microsomal triglyceride transfer protein (MTTP). Normally, insulin inhibits MTTP synthesis and promotes apo B100 degradation. In MASLD, however, insulin does not prevent formation of VLDL particles, even in post-prandial states when *de novo* lipogenesis is occurring. VLDL size increases in MASLD and prevents them from exiting the liver through endothelial sinusoids, resulting in hepatic fat accumulation (37).

One major implication of hepatic insulin resistance is its connection with vascular insulin resistance. A mouse model of liver-specific insulin receptor knockout subsequently developed insulin resistance in aorta and heart and endothelial dysfunction and inflammation (54). On the other hand, endothelial-specific insulin receptor deficiency did not affect systemic insulin sensitivity and plasma lipids, but accelerated atherosclerosis associated with endothelial NO synthase (eNOS) inactivation (55).

At a more molecular level, insulin resistance is driven by insulin-activated transcription factors such as sterol regulatory element-binding protein 1c (SREBP1c) and carbohydrate regulatory element-binding protein (ChREBP), which control expression of proteins involved in metabolic pathways such as fatty acid synthesis, glycolysis, and lipogenesis. Mechanistically, glucose has been shown to induce SREBP1c expression of lipogenic genes specifically (56) A study in rats demonstrated that diet-induced MASLD causes an increase in SREBP1c expression, which may be involved in inhibiting IRS2 expression and causing resistance to insulin (57) ChREBP also regulates genes involved in lipogenesis such as liver pyruvate kinase, fatty acid synthase, acetyl co-A carboxylase and hyperactivation of these pathways leads to enhanced production of fatty acids and triglycerides (58) Both SREBP1c and ChREBP are activated in MASLD (59, 60). As such, an enhanced overexpression and activation of these transcriptional regulators promotes the conversion of glucose into fatty acids, which drives steatosis and hepatic insulin resistance in a cyclic fashion that has systemic implications as aforementioned. Also, oxidative stress is an activator of SREBP1c in HepG2 cells and causes hepatic fat accumulation, which in turn causes an increase in ROS, indicating a detrimental cycle where hepatic fat accumulation and SREBP1c overexpression progressively cause lipotoxicity and worsen MASLD (61, 62).

### Oxidative stress in MASLD

It is known that oxidants are involved in the pathology of MASLD. Mitochondrial dysfunction and increased oxidants generation have been detected in liver tissues from patients with MASLD. Mitochondria regulate fat oxidation and energy production, and also generate oxidants through the electron transport chain. Obese insulin-resistant individuals showed upregulated hepatic mitochondrial respiration in the early stage, but this adaptive response disappeared in progression of MASLD to MASH. Obese patients with MASH displayed elevated hepatic oxidative stress (H_2_O_2_) due to lower mitochondrial respiration, causing disturbed insulin receptor signaling, oxidative DNA damage, and systemic inflammation such as increased serum IL-6 (63). Thus, increased oxidants lead to insulin resistance (64). Also, GSH/GSSG is significant lower in type 2 diabetic patients and insulin increased GSH/GSSG (65), suggesting insulin can reduce oxidative stress by controlling GSH redox status.

Oxidative stress arises from imbalances between oxidant-generation and antioxidant systems. Patients and animal models with MASLD/MASH show increased oxidative markers (66). Plasma antioxidants capacity and antioxidant enzymes such as superoxide dismutase and catalase were lower in the liver of MASLD patients (66).

Nicotinamide adenine dinucleotide phosphate oxidases (NADPH oxidase, NOXs) are also involved development of MASLD. Hepatic NOX1 expression is enriched in liver sinusoidal endothelial cells (LSEC) and NOX1 deletion attenuates liver injury and apoptosis in high-fat fed mice (67) NOX2 (gp91phox) contributes to the generation of oxidants by Kupffer cells and infiltration of macrophages in the liver. NOX2 deficiency protected mice from high-fat induced steatosis and insulin resistance (68). Hepatocyte-specific NOX4 deletion decreased liver injury, apoptosis, oxidative stress, and fibrosis in mice with diet-induced MASH (69). However, a recent report shows that NOX4 is essential for the adaptive response to prevent progression to MASH. Human hepatic NOX4 gene expression is upregulated with steatosis but decreased in advanced MASH. Hepatic NOX4 overexpression attenuates MASH and fibrosis in high-fat diet fed mice (70), indicating a protective role of NOX4-derived oxidants in the MASH model.

Oxidants and metabolic stress activate nuclear factor erythroid 2-related factor 2 (Nrf2). Nrf2 induces genes involved in glutathione synthesis, thioredoxin, iron homeostasis, thus, Nrf2 activation ameliorates oxidative stress. Nrf2 is downregulated in MASH and pharmacological activation of Nrf2 increased glutathione (GSH) levels and attenuates MASLD and fibrosis (71, 72).

The antioxidant glutathione (GSH) is a tripeptide, produced from glutamic acid, cysteine, and glycine in a two-step process catalyzed by glutamate-cysteine ligase (Gcl) and glutathione synthetase. The liver highly expresses the rate-limiting enzyme Gcl (73). Therefore, a main GSH production occurs in the liver although any cell can produce GSH. GSH levels are lower in the liver of MASLD patients and further decreased with the association of insulin resistance (74, 75). Hepatic GSH is decreased also in high-fat fed rats and diet-induced MASH mice (66, 71, 76). Data obtained from MASH patients and animal models indicate an association between the depletion of hepatic GSH and development of MASH.

**Figure 4:**
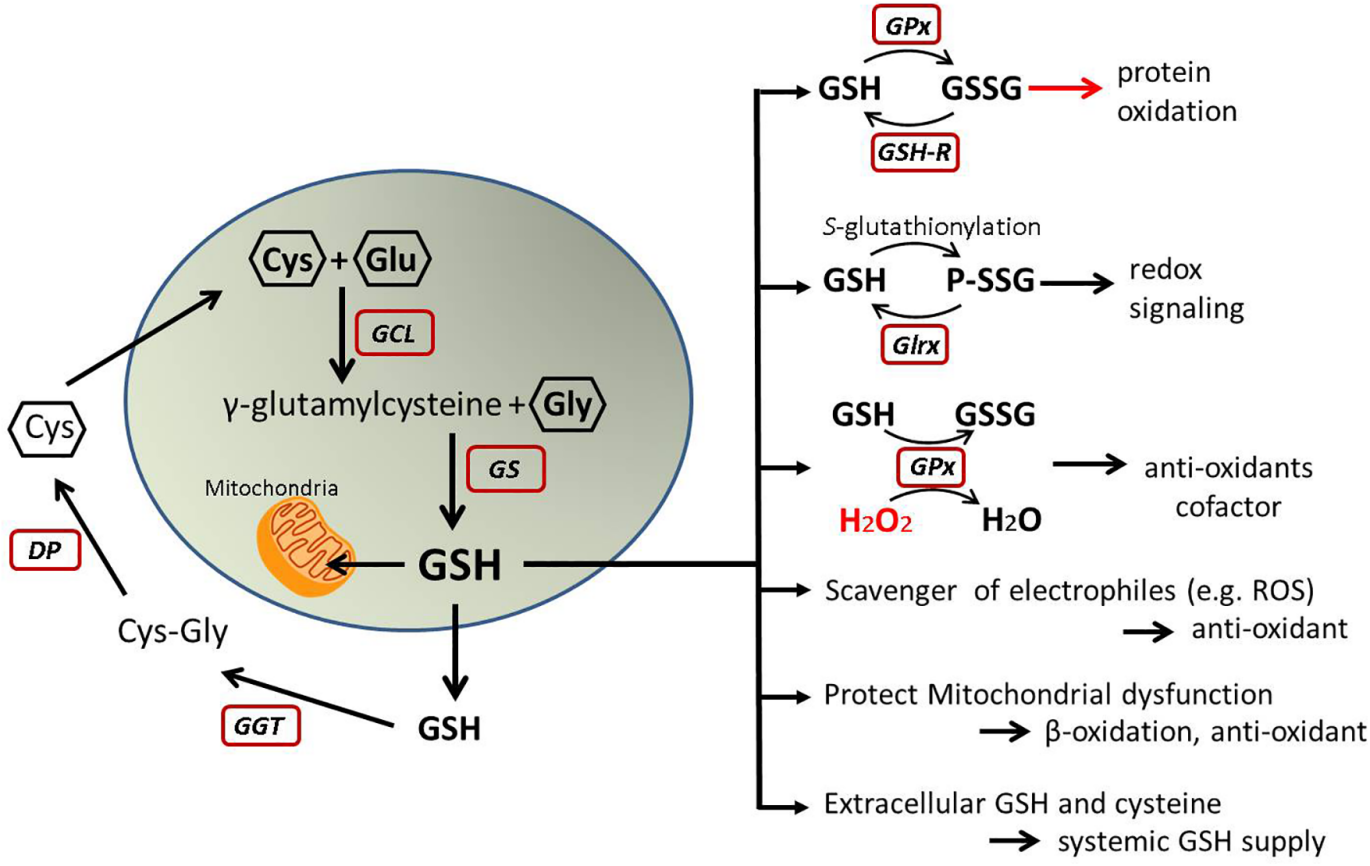
GSH metabolism and function. This illustrates GSH synthesis and recycles in the cells and its molecular functions. Oxidized GSH (GSSG) may cause oxidative stress, but other roles are protective from oxidative stress and help to maintain redox homeostasis. GSH, glutathione; GSSG, oxidized glutathione; Cys, cysteine; Glu, glutamic acid; Gly, glycine; GCL, glutamate-cysteine ligase; GS, glutathione synthetase; GGT, gamma-glutamyl transferase; DP, dipeptidases; GPx, glutathione peroxidase; GSH-R, glutathione reductase; Glrx, glutaredoxin; P-SSG, glutathionylated protein; H_2_O_2_, hydrogen peroxide; ROS, reactive oxygen species.

Since the liver is a major source of GSH production, it plays a role in the inter-organ homeostasis of GSH and cysteine (77–79) Therefore, lower GSH levels in the liver may affect the redox status in the heart or other organs. A clinical study shows plasma GSH level is significantly decreased in MASLD or MASH patients compared to healthy age-, sex-matched control (80). Lower plasma GSH is associated with cardiovascular risks (81). Dietary supplements which potentially increase plasma GSH improve cardiometabolic health in diabetic patients (82). Taken together, decreased hepatic GSH synthesis in MASLD may cause systemic depravation of GSH and increased oxidative stress, which is a major risk factor of CVD. (Figure 4)

Also, circulating GSH can be recycled. Gamma-glutamyl transferase (GGT) is an enzyme which recycles cysteine from extracellular GSH to promote replenishment of intracellular GSH. Elevated serum GGT is a reflection of oxidative stress and correlates with higher incidence of metabolic disease and CVD as shown in a Framingham Heart Study (83).

### Inflammation in MASLD

Immune cells activation in the liver contributes to MASLD progression (84–86). Inflammatory cytokines increase from adipose tissue in the context of MASLD. Among circulating cytokines, a meta-analysis indicates that CRP, IL-1β, IL-6, and TNF levels are associated with increased risk of MASLD (87). Uptake of lipids via macrophage scavenger receptor 1 induces a JNK-mediated pro-inflammatory response, increasing production of cytokines such as IL-6 and TNF (88). These are pro-inflammatory and pro-atherogenic cytokines causing CVD risk. A clinical study has shown that inhibiting IL-1β significantly reduces cardiovascular events (89). IL-6 deletion attenuates left ventricular hypertrophy and dysfunction induced by pressure overload, indicating IL-6 signaling is essential cardiac myocytes hypertrophy (90). Circulating levels of both IL-6 and high-sensitive C-reactive protein (hsCRP) are independently associated with risk of CVD (91, 92). Tumor necrosis factor (TNF*α*) is associated with insulin resistance and induces inflammatory cytokines. Thus, inflammation in the liver may cause systemic factors to connect MASLD and CVD.

Multiple populations of macrophages are in the liver and are thought to have pro-inflammatory roles. However, a specific population of human resident liver immune cells may express antioxidant activity and protect metabolic impairment in obesity by reducing oxidative stress (93).

Also, low-grade inflammation associated with MASLD comes from gut microbiota imbalance. High fat high sugar diet alters gut microbiota and increases bacteria and other organisms which produce LPS, pathogen-associated molecular pattern, and harmful metabolites, resulting in the activation of inflammatory pathways (94–96). Gut microbiota alteration (dysbiosis) was one of the risk factors relating to severity of MASLD and associated with inflammation, ballooning, and fibrosis in MASLD patients (97). Gut microbiota imbalance causes oxidative stress, systemic inflammation, and significant impacts on CVD (98). For example, LPS promotes pro-inflammatory status in atherosclerotic artery, leading plaque instability and thrombus formation (45). A gut bacteria-derived metabolite, trimethylamine N-oxide (TMAO), promotes form cell formation and atherosclerosis (99). Elevated circulating TMAO levels relate to CVD risk and mortality in multiple cohort studies as well as severity of MASLD (98, 100). Thus, gut microbiota influence pathology of CVD and MASLD. A meta-analysis has shown that microbiota intervention with probiotics and prebiotics improves inflammation, insulin resistance, dyslipidemia, obesity, liver injury in MASLD patients (101) and probiotics can suppress steatosis in high-cholesterol fed rabbits (102). Presumably, this treatment may reduce the incidence of CVD in MASLD patients.

In addition, secretion of microRNA (miRNA) is changed with MAFLD. The steatotic liver increased secretion of miRNA-containing small extracellular vesicles which promote form cell formation and atherosclerosis by inhibiting ABC1-mediated cholesterol efflux (43) or cause endothelial inflammation by activating NF-kB activity (103).

### Liver fibrosis and cirrhosis

During the chronic progression of MASLD, immune cells activation induces HSC activation and differentiation into myofibroblast-type cells, which produce extracellular matrix leading collagen accumulation. Liver fibrosis precedes cirrhosis with its associated complications such as liver failure and hepatocellular carcinoma (104–106).

Liver fibrosis in the setting of MASLD is the strongest predictor of prognosis and mortality. Fibrosis severity has been linked to adverse metabolic outcomes, cardiovascular disease, mortality, and liver-related morbidity (107–110). For example, A Korean population study showed MASLD patients with advanced liver fibrosis measured via the BARD score had higher incidents of heart failure and cardiovascular mortality when they compared MASLD patients without severe fibrosis (111). Similarly, fibrosis scoring systems NFS (NAFLD Fibrosis Score) and FIB-4 (Fibrosis 4 Index) were found to correlate with CVD mortality (112) Therefore, it is important to reverse liver fibrosis or block the fibrotic process in MASLD to reduce mortality and comorbidity of CVD.

One mechanistic target that has been identified for treating MASLD is fibroblast growth factor 21 (FGF-21), which is involved in ferroptosis, a process that in the liver has recently gained attention due to its implications in metabolic disease (113). Ferroptosis is an iron-dependent mechanism of programmed cell death, which is achieved via oxidative stress, and has significant implications in the development of hepatic fibrosis (114, 115). While FGF-21 overexpression protects hepatocytes from being damaged in mitochondria-driven oxidative processes, the lack of FGF-21 induces iron-overloaded ferroptosis, driving hepatic fibrosis (116). A clinical trial assessing the efficacy of Pegozafermin, an FGF-21 analogue, has shown improvements in fibrosis, opening the door to the possibility of attenuating CVD in the context of advanced MASLD (117).

When liver fibrosis further progresses, and chronic MASLD leads to liver cirrhosis, one consequence that manifests is portal hypertension, or hypertension of the portal vein, which has systemic implications and can affect the heart (118). Specifically, cirrhosis and portal hypertension can affect heart function, leading to a condition known as cirrhotic cardiomyopathy. The structural changes of cirrhotic liver and low endothelial NO production cause portal hypertension. The physiological response to elevated pressures in the portal vein is systemic vasodilation, which is followed by an activation of the renin-angiotensin-aldosterone system and sympathetic nervous system to maintain normal blood pressures, and concomitant increase in blood volume, all of which drive cirrhotic cardiomyopathy (25, 119).

Cirrhotic cardiomyopathy is a complex pathology combined systolic dysfunction (low ejection fraction) and diastolic dysfunction (impaired ventricle relaxation) according to cirrhotic cardiomyopathy consortium criteria (2019) (120) and can be explained by morphological changes in the heart, including dilation of left ventricle and thickening of the septum, among other examples of cardiac remodeling, which are features of cardiomyopathy (26, 121).

NO from endothelial NO synthase (eNOS, NOS3) is normally cardioprotective. However, in the decompensated liver cirrhosis, increased inflammatory cytokines (e.g., TNF, IL-1) stimulate inducible NO synthase (iNOS, NOS2) which can worsen cardiac function. In a cirrhotic cardiomyopathy animal model, iNOS expression, not eNOS, was upregulated and the NOS inhibitor improved cardiac muscle contractility (122). Notably, iNOS may produce harmful superoxide and peroxynitrite under certain conditions (123). Therefore, activated iNOS causes dysfunction of cardiac proteins (124).

As such, the dysregulation of portal vein blood pressures, inflammation, and oxidative stress are factors where the hepato-cardiac axis is impacted in the context of advanced MASLD, driving cardiovascular disease.

Evidence of liver fibrosis and abnormalities using MRI-derived iron-corrected T1 mapping (cT1) imaging technique is associated to CVD, in particular atrial fibrillation and heart failure, independently of other cardiovascular risk factors (125). This type of new imaging techniques open the door to non-invasive screening techniques to detect MASLD-related cardiovascular risks in early stages, leading to more widespread screening and timely diagnosis, potentially steering patients away from CVD.

Accumulation of hepatic fat with its broad consequences are also dictated by genetic polymorphisms, notably of patatin-like phospholipase domain-containing protein 3 (PNPLA3) and transmembrane 2 superfamily member 2 (TM6SF2) genes. In mice, abundance of the PNPLA3 I148M variant accumulates in lipid droplets in the liver, resulting in a fat accumulation. The subsequent knockdown of this gene diminished TG levels in the liver, showing its potent and isolated effect (126). The same rs738409 (I148M) polymorphism of the PNPLA3 gene is associated with the metabolic syndrome and insulin resistance in those who have MASLD (127). In the patients with diabetes who have an intermediate FIB-4 score for MASLD, this SNP has been linked to a high risk of cirrhosis, comparable to those with a higher FIB-4 score (126, 128). As such, genetic polymorphisms could be used to stratify MASLD patients more accurately, potentially preventing the cardiovascular consequences associated with progression of steatosis to cirrhosis. TM6SF2 also has implications in MASLD; studies have shown that it is required for hepatic VLDL secretion, whereas knocking it out in mice prevents hepatic TG from being packaged and exported as apolipoproteins, resulting in a 3-fold increase in the liver TG level (129). The same concept was elucidated ex vivo with human liver samples (130). Integrating genetic variability as a factor for diagnosis and treatment of MASLD may provide valuable information on how prone an individual may develop steatosis. Taken together, using genetic information to aid the classification of MASLD stages may be helpful to prevent cardiovascular complications before their development.

## Discussion

**Figure 5:**
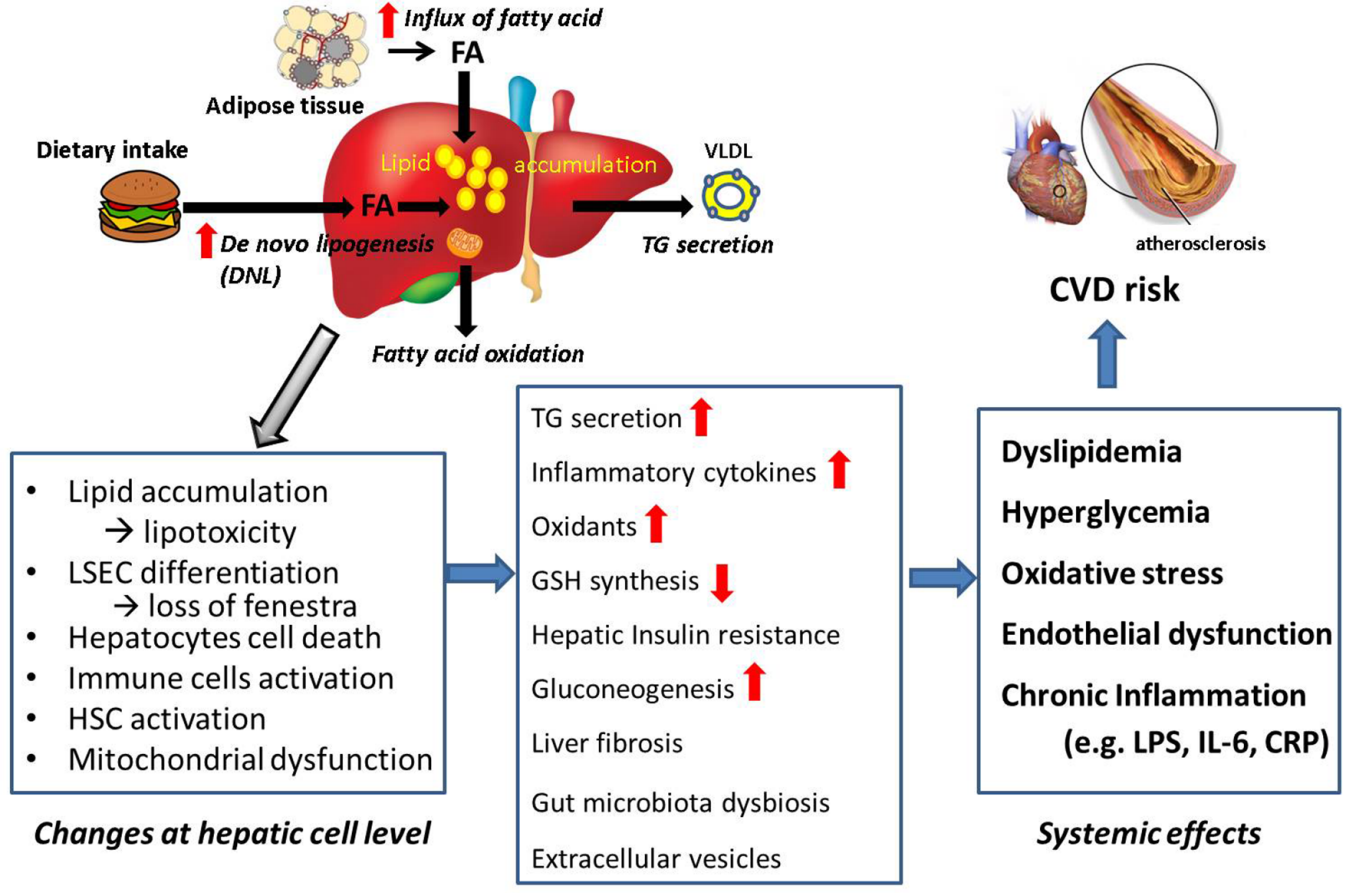
Summary: connection from MASLD to CVD. Lipid accumulation causes morphological and physiological changes at cellular levels in the liver, leading systemic effects which are CVD risk factors. Summary of the review is shown.

We reviewed how the hepatic pathological changes lead to increased CVD in MASLD patients. Metabolic changes lead to elevated levels of circulating lipids, glucose, and insulin, which contribute to atherosclerosis and increase the risk of CVD. In individuals with MASLD, the liver releases systemic factors such as inflammatory cytokines, LPS, and extracellular vesicles. These factors cause endothelial dysfunction and atherosclerosis, and further increase CVD risk. Additionally, decreased levels of GSH in the liver can exacerbate systemic oxidative stress, adding another layer of risk for cardiovascular incidents. Figure 5 is a summary of these processes. Furthermore, we discuss therapy and future directions.

### Antioxidants therapy and beyond

Excess oxidants or oxidative stress are involved in progression of MASLD as well as CVD. However, clinical trials of antioxidant vitamins (ascorbic acid, α-tocopherol, β-carotine) did not prove efficacy to treat or prevent CVD (131, 132). Interestingly, a clinical study of dietary intake of antioxidants by food demonstrated that vitamin E (*α*-tocopherol) was effective to reduce incidence of CVD (133). Also, cliniacal trials show vitamin E improved hepatic function in MASH patients without diabetes, but failed to improve in pediatric MASLD (134–136). Currently, vitamin E is approved to treat only non-diabetic adult patients with biopsy-proven MASH for short term treatment. The safety of high dose vitamin E is a concern (137). In addition, Nrf2 activator may enhance antioxidant response, and polyphenols, flavonoid, and metformin can improve mitochondrial dysfunction to reduce oxidative stress (138, 139). However, clinical studies and trials have not approved any antioxidant therapy for either MASLD or CVD.

Reactive oxidant species (ROS) generate oxidized phospholipids. Oxidized phospholipids further accumulate ROS and induce mitochondrial dysfunction in hepatocytes. It is shown that neutralizing antibody to oxidized phospholipids improved a mouse model of MASH, reducing steatosis, inflammation, and fibrosis (140). Therefore, targeting oxidized phospholipids, not ROS itself, can be therapeutic.

As mentioned above, there is an association between the depletion of hepatic GSH and development of NASH. Increased GSH by antioxidant compound such as N-acetylcysteine can reduce oxidative stress and inflammation in animal models of MASLD (141). A few clinical studies indicate that direct GSH administration by oral or intravenous injection improved liver enzymes in patients with MASLD (79). Sublingual form of GSH was more effective to increase total GSH and plasma vitamin E level compared to oral GSH or N-acetylcysteine (142).

The common notion is that antioxidants scavenge oxidants and are beneficial to our health, but ROS are essential to mediate cellular signaling by generating oxidative modification of proteins. Therefore, excess antioxidant to eliminate oxidants may disturb physiological responses that require reactive oxygen species (ROS) and, in turn, be harmful (143, 144). This must be one of the reasons antioxidants do not necessarily improve pathological conditions.

In addition, GSH serves as scavenger of oxidants but exists in different forms inside cells. Excess oxidants and glutathione peroxide generate oxidized glutathione (GSSG, oxidized form), whereas glutathione reductase reverses it to GSH (reduced form). In diet-induced MASLD, hepatic GSH decreases, whereas GSSG is elevated, and GSSG sensitizes hepatocytes to TNF-induced cytotoxicity (145). GSSG accumulation or a higher ratio of GSSG/GSH causes oxidative modification on protein thiols or cysteine (Cys), a mechanism called *S*-glutathionylation (R-SSG). This oxidative modification may alter cellular signaling; for instance, it inhibits SirT1 and NFkB pathways, and induces apoptosis (143, 146–149). Also, phosphatase and tensin homolog (PTEN) is activated by S-glutathionylation in the liver of diet-induced NAFLD (150). PTEN is a negative regulator of insulin signaling and its activation may promote insulin resistance (151).

**Figure 6:**
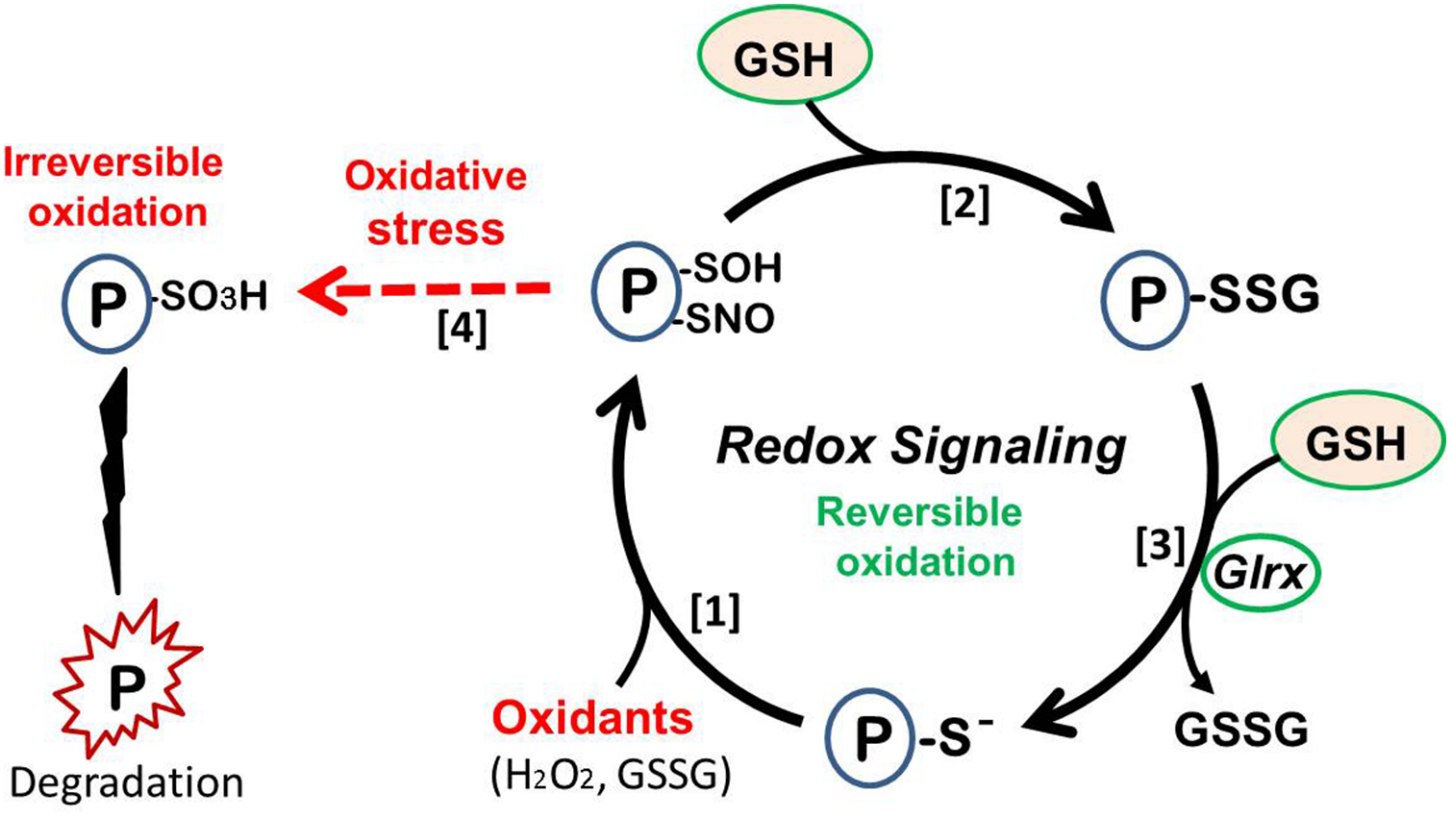
Redox signaling. (1) Protein thiol oxidation, leading to sulfenylation (P-SOH) or nitrosylation (P-SNO). (2) React with abundant cellular GSH to form protein S-glutathionylation (P-SSG, protein-bound GSH). (3) S-glutathionylaton can be reduced by glutaredoxin-1 (Glrx). This cycle generates reversible protein function and causes cellular signaling, preventing irreversible damage of the protein. (4) Strong oxidative stress causes irreversible oxidation (e.g., sulfonylation; P-SO3H), resulting in protein dysfunction or degradation (left side).

Furthermore, the small enzyme glutaredoxin-1 (Glrx) catalyzes the reversal of R-SSG. Thus, S-glutathionylation is reversible, and Glrx can activate SirT1 and inhibit apoptosis (152). In MASLD/MASH livers, Glrx expression decreases and the amount of R-SSG (glutathionylated proteins) increases (145, 153). When this redox cycle is disturbed by lower Glrx activity, oxidized proteins can become irreversibly-oxidized proteins which turn to be dysfunctional or degraded. Therefore, Glrx prevents protein thiols from permanent oxidation and maintains protein function under the presence of oxidants. (Figure 6) Proteomics analysis of plasma proteins from young adults indicates thiol oxidation is progressive with cardiovascular risks. Irreversible oxidation increased and Glrx expression decreased in patients with reported cardiovascular event (154).

Mice lacking Glrx develop MASLD (fatty liver, obesity, dyslipidemia) with a regular diet, while adenoviral Glrx gene transfer activates SirT1 and reverses steatosis (147). Pre-clinical studies using an adeno-associated viral vector demonstrated that administration of Glrx specifically targeting hepatocytes attenuates fibrosis and inflammation in the liver of diet-induced MASH mice (153). Importantly, Glrx enzyme activity requires GSH and glutathione reductase. Therefore, GSH is not only an antioxidant itself but also an essential cofactor for the reducing enzyme. Glycine-based treatment increases GSH synthesis, fatty acid oxidation, and attenuates diet-induced MAFLD, suggesting that enhancing hepatic GSH synthesis is therapeutic for the patients with MASLD (155). Glrx is also protective in the heart mainly by inhibiting apoptosis (156, 157), highlighting its importance in alleviating the cardiovascular consequences that may occur in MASLD/MASH (158).

### Therapy for MASLD and future direction

There are different pharmacological approaches to improve MASLD and multiple clinical trials have been conducted. There are summary of major current drugs under clinical trials or pre-clinical studies in Table 1. The therapy for MAFLD is reviewed more in details elsewhere (173, 174).

**Table 1:** Overview of preclinical and clinical therapies for MASLD. The table includes treatments currently in use and experimental therapies under investigation.

	Drug therapy	Study type	Target mechanism	Key findings
Clinical trials	Acarbose	Clinical (Phase IV)	Alpha-glucosidase inhibitor	In patients with CAD and impaired glucose tolerance significantly reduced incident diabetes and facilitated regression to normoglycemia (159)
	Elafibranor	Clinical (phase III)	PPAR-α/δ agonist	Lessened hepatic fibrosis, and improved cardiometabolic risk factors such as better glycemic control and lower LDL cholesterol, (160)
	Obeticholic Acid (OCA)	Clinical (phase III)	Farnesoid X receptor agonist	Reduces liver fibrosis but transiently increases LDL cholesterol levels (161)
	Nicotinamide Riboside + Pterostilbene (NRPT)	Clinical (phase II)	Anti-inflammatory and antioxidant	Reduced markers of hepatic inflammation in MASLD. Decreased ALT, GGT, and ceramide 14:0 (162)
	GLP-1 RA + GIP RA	Clinical (phase II)	GLP-1 and GIP receptor agonists	In patients with T2DM lowered body weight and imoroved glycemic control (163)
	Resmetirom	Clinical (phase III)	Thyroid hormone receptor beta (TRβ) agonists	Decreases hepatic fibrosis and LDL cholesterol levels. First FDA approved drug for MASLD (164)
	GLP-1 RA + GCG RA	Clinical (phase II)	GLP-1 and glucagon receptor agonists	In patients with MASLD decreased hepatic fat and inflammation, and body weight. (165)
	SGLT2 Inhibitors (e.g., Empagliflozin)	Clinical (phase II)	SGLT2 inhibition	Hepatic fat decreases in relation to decreases in body weight and improvement in insulin sensitivity (166)
	Semaglutide	Clinical (phase II & III)	GLP-1 receptor agonist	Did not resolve hepatic fibrosis or MASH, but is known to lower weight and improve cardiovascular risk factors (167, 168)
	G-protein-coupled receptor 40 agonist	Clinical (phase I)	G-protein-coupled receptor 40 activation	Improves glucose metabolism in patients with T2DM by facilitating secretion of hormones such as insulin, glucagon, GLP-1, GIP, PYY (169)
	Dasatinib + Quercetin	Clinical (phase I & II)	Senolytic approach	Reverses age-related scenescence and ameliorates inflammation in adipose. Currently in trial to examine effects on liver fibrosis in MASLD (170)
Preclinical	Metformin + Resveratrol + Rapamycin	Preclinical	Nutritional reprogramming	Combined therapy dampened the response of liver proteome and mitochondria to intake of energy and macronutrients in mice (171)
	N-Acetylcysteine (NAC)	Preclinical	Antioxidant	Prevented maternal weight gain with HFD during pregnancy, and reduced hepatic DAG and TG levels, and led to favorable metabolic outcomes in offspring as well (172)
	Glutaredoxin-1 (Glrx)	Preclinical	Thiol transferase (reducing oxidative modification)	AAV-mediated administration into hepatocytes suppressed fibrosis and inflamation in diet-induced MASH mice (153)
	Antibody (E06) to oxidized phospholipids (OxPLs)	Preclinical	Neutralize oxidized phospholipids	Neutrzlizing antibody (E06-scFv) overexpressing mice attenuated steatosis, inflammation, fibrosis, progression to hepatocellular caricinoma in MASH model (140)

Anti-diabetic drugs have been used for metabolic impairment. Metformin improves glucose metabolism and reduce body weight, and likely reduce CV risk in diabetic patients. However, clinical trials have not shown significant effects on steatosis, MASH, and fibrosis (175). Pioglitazone, peroxisome proliferator-activated receptors (PPAR)-gamma agonist, improves insulin resistance and hepatic steatosis, but its effect on liver fibrosis is unclear. Pioglitazone may be considered for use in patients with T2DM and with biopsy-proven MASH. Its effects on CVD are also unclear although some trials show a protective effect (175). Glucagon-like peptide 1 (GLP-1) agonist (e.g., Semaglutide) decreases steatosis, obesity, insulin sensitivity, and CV risks. Sodium glucose cotransporter 2 (SGLT-2) inhibitor is also ant-diabetic drug. SGLT-2 inhibitor suppresses oxidative stress, ER stress, and inflammation, and reduces steatosis and fibrosis in animal studies, but improvement of fibrosis was not consistent in human clinical studies (47).

The preventive effects on hepatic fibrosis would be beneficial to reduce CVD associated MASLD. PPAR*α*/*Δ* agonist (Elafibranor) could suppress fibrosis and CVD risk factors. Farnesoid X receptor against (Obeicholic acid) also reduces fibrosis in MASH (110). Statin, which inhibits cholesterol synthesis via HMG-CoA reductase, inhibit hepatic fibrosis and progression of MASLD independent on diabetes (176). As we mentioned before, a FGF-21 analogue (Pegozafermin) shows significant effects on liver fibrosis in biopsy-proven MASH patients in a clinical trial (117).

Recently, FDA approved the first drug specifically intended to treat patients with MASLD. Clinical trials showed the efficacy of Resmetirom, a thyroid hormone receptor (THR) β agonist, for MASH with fibrosis (164). Thyroid hormone reduces steatosis by stimulating autophagy, mitochondrial biogenesis, fatty acid oxidation, and controls cholesterol synthesis (177), and attenuates oxidative stress and inflammation as shown in diet-induced MASH mice (178). Its effect of lowering hyperlipidemia might have an impact on atherosclerotic complications. Despite the potential to attenuate CVD in patients with MASLD, thyroid hormone may show adverse effects in the heart such as tachycardia and arrhythmia. The heart mainly expresses THRα isoform and the liver expresses THRβ, therefore, it was critical to generate a THRβ specific agonist (179) Clinical studies in future will reveal how the drug can prevent CVD associated with MASLD.

There are more lines of pre-clinical and clinical studies. Glutaminase-1 (GLS1) which involved in glutamine metabolism is overexpressed in the liver of MASH patients. GLS1 inhibitor in pre-clinical models reduces steatosis and oxidative stress (180). Nicotinamide riboside and pterostilbene, known as a supplement Basis, reduce markers of hepatic inflammation in MASLD (162). The combination therapies of these drugs are recommended to hit several targets or pathways to treat MASH (181).

CVD risks arise from multiple factors. Recently, the American Heart Association updated the risk prediction equation PREVENT (AHA Predicting Risk of CVD Events) including kidney function (182). Given the prevalence MASLD and the direct effect of hepatic pathophysiological mechanisms to cardiovascular health, the liver-cardiovascular axis can be considered in future equations. Hepatic factors relating to MASLD such as plasma levels of ALT, AST, GSH, CRP, and hepatic CT can be included to improve the long-term risk prediction of CVD.

## Conclusion

MASLD starts with accumulation of excess lipids in the liver. Fatty acid intake and *de novo* lipogenesis exceed disposal of lipids from the liver, and lipotoxicity triggers hepatocyte injury, immune cell activation, mitochondrial dysfunction, leading to increased oxidants generation and production of inflammatory cytokines. LSECs lose unique fenestra leading to impairments in the hepatic transport of macromolecules. HSCs are activated to produce collagens. Impaired insulin signaling in the liver leads to hyperglycemia and hyperinsulinemia, and upregulated insulin further activates *de novo* lipogenesis pathway. Excess oxidants generation lowers antioxidant capacity and decreases GSH level in the liver.

These pathological changes in the liver result in dyslipidemia, hyperglycemia, increased circulating inflammatory molecules, gut microbiota imbalance, and decreased plasma GSH. All the systemic effects promote atherosclerosis and CVD incidents. Thus, MASLD is a risk factor of CVD.

We emphasize that oxidative stress is a key connection between progress of MASLD and CVD. Oxidants are not only harmful to cells, but also alter protein function by post-translational thiol oxidation. Therefore, for example, administration of reducing enzyme Glrx can be helpful to battle with oxidative stress and prevent irreversible oxidation of proteins in case of advanced MASLD or CVD. Anti-oxidant therapy is not merely eliminating radicals. It is important keep the homeostasis of redox signaling.
